# Detection of Pancreatic Carcinomas by Imaging Lactose-Binding Protein Expression in Peritumoral Pancreas Using [^18^F]Fluoroethyl-Deoxylactose PET/CT

**DOI:** 10.1371/journal.pone.0007977

**Published:** 2009-11-24

**Authors:** Leo Garcia Flores, Susanna Bertolini, Hsin Hsin Yeh, Daniel Young, Uday Mukhopadhyay, Ashutosh Pal, Yunming Ying, Andrei Volgin, Aleksandr Shavrin, Suren Soghomonyan, William Tong, William Bornmann, Mian M. Alauddin, Craig Logsdon, Juri G. Gelovani

**Affiliations:** 1 Department of Experimental Diagnostic Imaging, Center for Advanced Biomedical Imaging Research (CABIR), The University of Texas MD Anderson Cancer Center, Houston, Texas, United States of America; 2 Department of Cancer Biology, The University of Texas MD Anderson Cancer Center, Houston, Texas, United States of America; 3 Department of Experimental Therapeutics, The University of Texas MD Anderson Cancer Center, Houston, Texas, United States of America; Genentech, United States of America

## Abstract

**Background:**

Early diagnosis of pancreatic carcinoma with highly sensitive diagnostic imaging methods could save lives of many thousands of patients, because early detection increases resectability and survival rates. Current non-invasive diagnostic imaging techniques have inadequate resolution and sensitivity for detection of small size (∼2–3 mm) early pancreatic carcinoma lesions. Therefore, we have assessed the efficacy of positron emission tomography and computer tomography (PET/CT) imaging with *β*-O-D-galactopyranosyl-(1,4′)-2′-deoxy-2′-[^18^F]fluoroethyl-D-glucopyranose ([^18^F]FEDL) for detection of less than 3 mm orthotopic xenografts of L3.6pl pancreatic carcinomas in mice. [^18^F]FEDL is a novel radioligand of hepatocarcinoma-intestine-pancreas/pancreatitis-associated protein (HIP/PAP), which is overexpressed in peritumoral pancreatic acinar cells.

**Methodology/Principal Findings:**

Dynamic PET/CT imaging demonstrated rapid accumulation of [^18^F]FEDL in peritumoral pancreatic tissue (4.04±2.06%ID/g), bi-exponential blood clearance with half-lives of 1.65±0.50 min and 14.14±3.60 min, and rapid elimination from other organs and tissues, predominantly by renal clearance. Using model-independent graphical analysis of dynamic PET data, the average distribution volume ratio (DVR) for [^18^F]FEDL in peritumoral pancreatic tissue was estimated as 3.57±0.60 and 0.94±0.72 in sham-operated control pancreas. Comparative analysis of quantitative autoradiographic images and densitometry of immunohistochemically stained and co-registered adjacent tissue sections demonstrated a strong linear correlation between the magnitude of [^18^F]FEDL binding and HIP/PAP expression in corresponding regions (r = 0.88). The in situ analysis demonstrated that at least a 2–4 fold apparent lesion size amplification was achieved for submillimeter tumors and to nearly half a murine pancreas for tumors larger than 3 mm.

**Conclusion/Significance:**

We have demonstrated the feasibility of detection of early pancreatic tumors by non-invasive imaging with [^18^F]FEDL PET/CT of tumor biomarker HIP/PAP over-expressed in peritumoral pancreatic tissue. Non-invasive non-invasive detection of early pancreatic carcinomas with [^18^F]FEDL PET/CT imaging should aid the guidance of biopsies and additional imaging procedures, facilitate the resectability and improve the overall prognosis.

## Introduction

The estimated number of new cases of pancreatic cancer in the United States in 2009 is 42,470 and the estimated number of deaths is 35,240 [1]. Although, pancreatic cancer accounts for only 2% of all malignancies, it is the fourth most common cause of cancer death in the United States. Such a poor prognosis for patients with pancreatic cancer is because 80–90% of patients have unresectable disease at the time of diagnosis [2]. It is widely accepted that the overall and disease-free survival of patients with pancreatic carcinoma are related to the possibility of complete macroscopic R0 resection [3]. Prognosis is also related to tumor size (<30 mm), nodal involvement and resection margin status [4]. Therefore, early diagnosis of pancreatic carcinoma with highly sensitive diagnostic imaging methods is very important and could save lives of many thousands of patients, because early detection of small pancreatic cancers is likely to increase resectability rates.

Due to inadequate resolution and sensitivity of ^18^F-FDG PET/CT imaging (∼5 mm in plane) for detection of small size (∼2–3 mm) early pancreatic carcinoma lesions [5], we have focused on the development of imaging agents for visualization of tumor-induced biomarkers that are over-expressed in the peritumoral pancreratic tissue. Such an approach should generate the “lesion size amplification” effect and result in at least 3 to 4 fold increase in the apparent lesion size. Such an approach could potentially improve the overall detectability of pancreatic tumor lesions at early stages of their development, improve their respectability, and the overall prognosis.

Among pancreatic tumor biomarkers produced in peritumoral reactive pancreatic tissue, the hepatocarcinoma-intestine-pancreas/pancreatitis-associated protein (HIP/PAP) was found to be over-expressed more than 130-fold in pancreatic acinar cells, as compared to normal pancreas [6]. In contrast, only a 9-fold increased expression of HIP/PAP protein was observed in acinar cells in chronic pancreatitis. Also, fragments of HIP/PAP protein are immunodetectable in blood and their levels correlate with the severity of pancreatic inflammation and pancreatic carcinoma size [7]. Furthermore, HIP/PAP protein is a promising target for the development of imaging agents, because it is also overexpressed in hepatocellular and cholangial carcinomas within the tumor cells [8].

The HIP/PAP protein is a 16 kD secreted protein, which belongs to the group VII of a family of proteins that contain a C-type lectin like domain, according to Drickamer's classification [9], which binds carbohydrates, and that it is also known as the “lactose-binding protein” (for review [10]. Previous biochemical studies demonstrated that GST-purified recombinant HIP/PAP protein has a very high affinity to D-lactose, but insignificantly to α-D-glucopyroanose, α-L-fucose, α-D-galactopyranose, or N-acetyl-β-D-glucosamine [11]. We hypothesized, that the HIP/PAP (the lactose-binding protein) should bind minimally modified ^18^F-labeled lactose analogues with high affinity, and that this “receptor-ligand” type of an imaging approach should allow for visualization of peritumoral infiltrated pancreatic tissue that 130-fold over-expresses the HIP/PAP protein, as compared to normal pancreatic tissue. Furthermore, our enthusiasm was supported by a previous report on biodistribution of *β*-O-D-galactopyranosyl-(1,4′)-2′-[^18^F]fluoro-2′-deoxy-D-glucopyranose ([^18^F]FDL) in mice, which demonstrated that [^18^F]FDL was not accumulating in any of the major organs and was rapidly cleared from the circulation by renal clearance and appeared in urine as non-metabolized parent compound, [^18^F]FDL [12]. Recently, we reported on the optimized radiosynthesis of *β*-O-D-galactopyranosyl-(1,4′)-2′-deoxy-2′-[^18^F]fluoroethyl-D-glucopyranose ([^18^F]FEDL) [13].

In this paper we demonstrate the efficacy of microPET/CT with [^18^F]-FEDL for detection of early microscopic pancreatic carcinoma lesions in a bioluminescent variant of an orthotopic pancreatic carcinoma xenograft model in mice. The results of non-invasive *in vivo* PET/CT imaging were validated by comparative *in situ* autoradiographic and immunohistochemical analyses. We conclude, that non-invasive detection of early pancreatic carcinomas with [^18^F]FEDL PET/CT imaging should aid the guidance of biopsies and additional imaging procedures, facilitate the resectability and improve the overall prognosis.

## Results

### Orthotopic Pancreatic Tumor Xenograft Model

**Figure 1:**
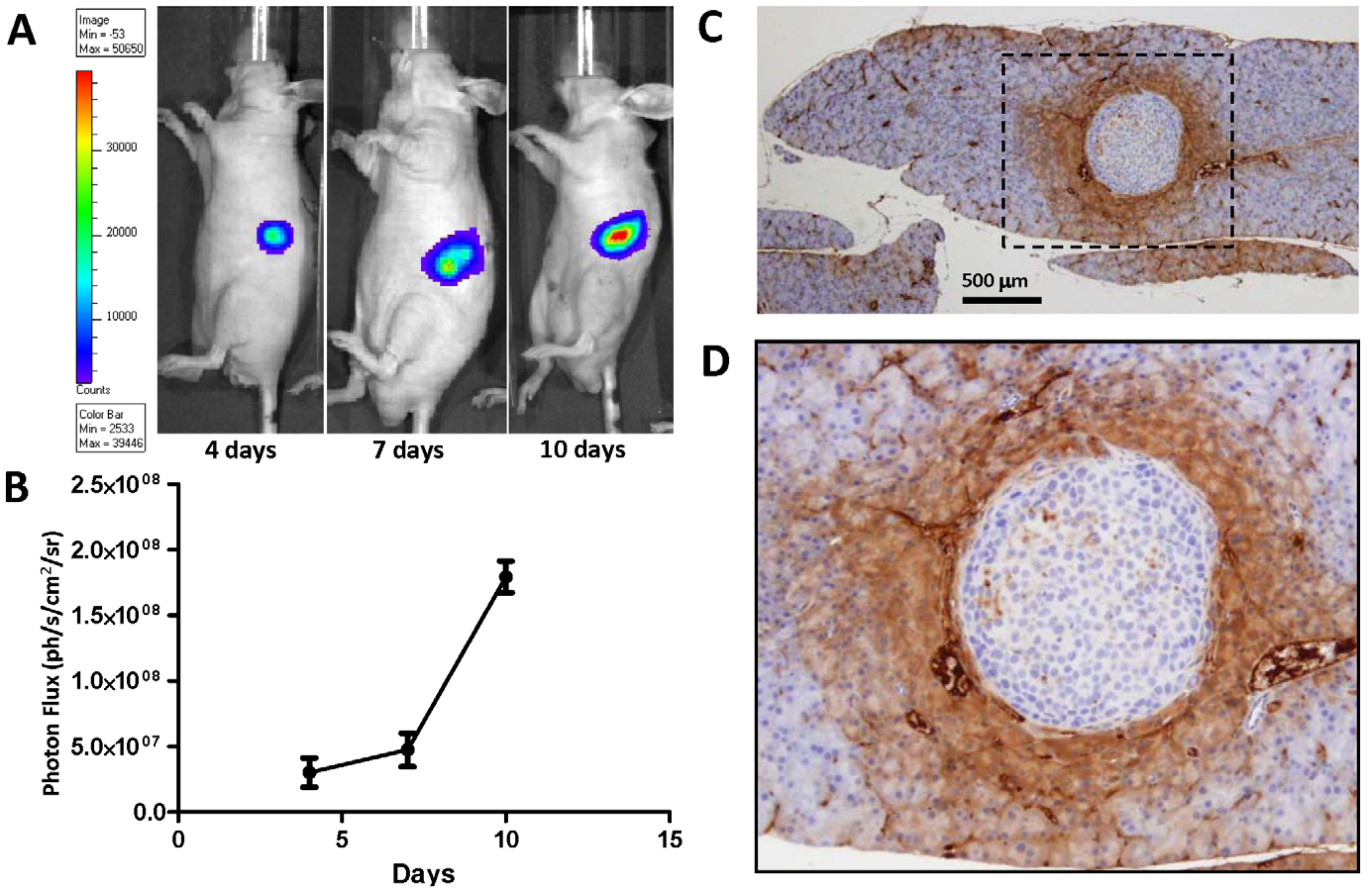
An orthotopic pancreatic tumor xenograft model in mice. (**A**) Bioluminescence images of mice with orthotopic tumors obtained at 4, 7, and 10 days post intra-pancreatic injection of L3.6pl/GL+ human pancreatic carcinoma cells. (**B**) Growth dynamics of the orthotopic L3.6pl/GL+ tumors based on the photon flux measured from BLI images obtained from the left mid-abdominal area. (**C**) HIP/PAP expression in peritumoral pancreatic acinar cells and microvesels (×20; bar = 500 µm); the black dotted line defines the area shown at higher magnification (×40) in panel (**D**).

In the Group I mice (N = 12), non-invasive bioluminescence imaging (BLI) was used for monitoring the growth of orthotopic pancreatic carcinoma xenografts. Distinct bioluminescence imaging (BLI) signal was detectable in the area consistent with the site of the L3.6pl/GL+ cell injection already on day 4 (**Fig. 1A**) and increased exponentially by day 10 post injection (**Fig. 1A,B**). Based on the results of BLI, PET/CT imaging studies in Group I, subsequent PET/CT imaging studies with [^18^F]FEDL were performed 7 days after injection of L3.6pl/GL+ cells, when the tumor diameter was 1.8±0.9 mm. However, based on the IHC analysis of pancreatic tissue sections, the apparent diameter of the lesion based on the expression of HIP/PAP in peritumoral pancreatic tissue ranged from 2 mm for sub-millimeter sized tumors (**Fig. 1C**) to almost a half of the pancreas (10–12 mm) for tumors of 2–3 mm in diameter. In all cases, at least a 2–4 fold amplification of the apparent tumor lesion size was observed, based on the extent of HIP/PAP expression in the peritumoral pancreas.

### PET/CT with [^18^F]FEDL

**Figure 2:**
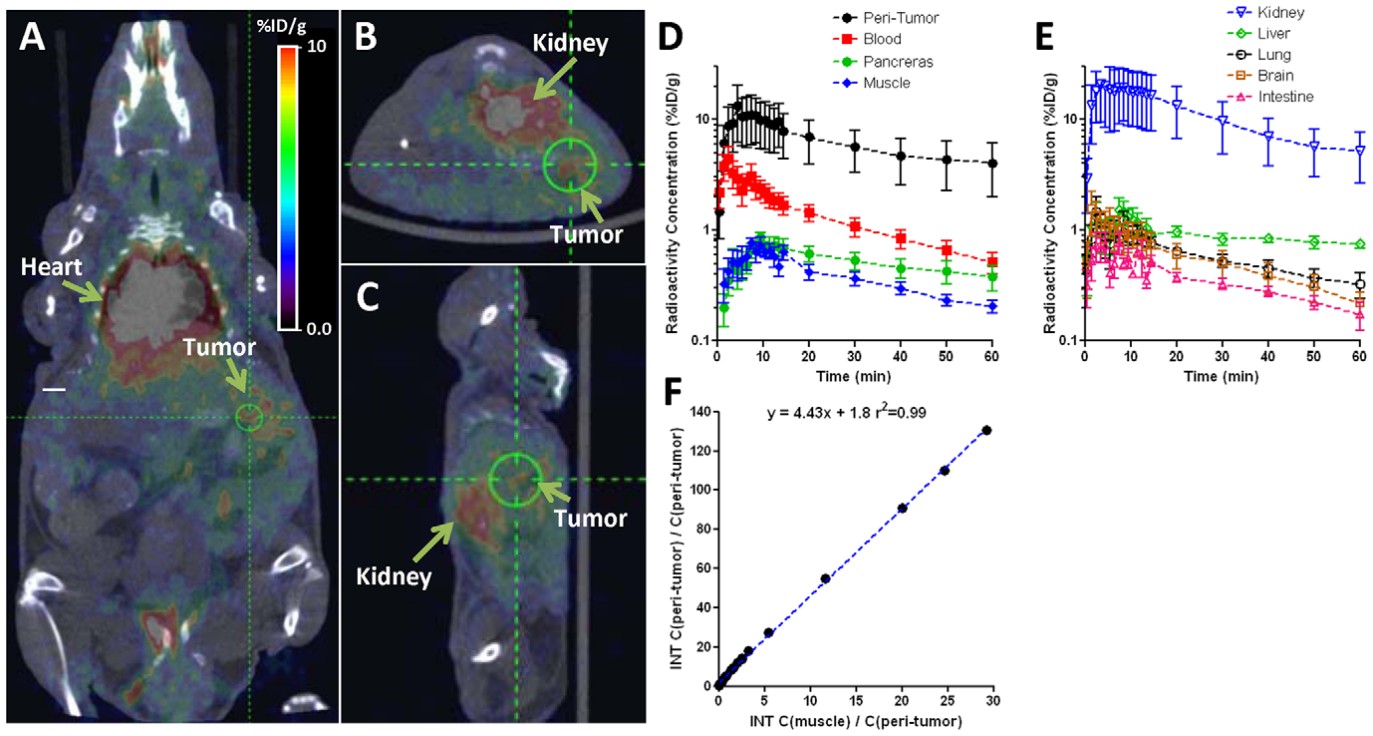
In vivo dynamic PET/CT imaging with [^18^F]FEDL. (**A**) Coronal, (**B**) axial, and (**C**) sagittal PET/CT images obtained at 60 min after i.v. administration of [^18^F]FEDL in a representative animal. (**D**) and (**E**) – time-activity curves of [^18^F]FEDL-derived radioactivity concentration in peritumoral pancreas and in different organs and tissues; points show means, bars – standard deviations. (**F**) Logan plot analysis to quantify the distribution volume ratio (DVR) of [^18^F]FEDL in peritumoral pancreas using muscle as a reference tissue for the representative animal shown in panels **A–C**.

Dynamic PET imaging demonstrated a rapid accumulation of [^18^F]FEDL in the area of L3.6pl/GL+ tumor growth with characteristically concentric or a horseshoe pattern (**Fig. 2A–C**), which corresponds to the pancreatic tail adjacent to the visceral surface of the spleen and anterior to the upper pole of the left kidney. Model-independent graphical analysis of dynamic PET imaging data (Logan plot) using muscle as the reference tissue devoid of HIP/PAP protein expression, the average distribution volume ratio (DVR) for [^18^F]FEDL in peritumoral pancreatic tissue was 3.57±0.60 and with the binding potential (BP) of 2.57±0.60 (**Fig. 2F**). In sham-operated control animals, the DVR for [^18^F]FEDL in the pancreas was 0.94±0.72. The differences in DVR and BP between tumor-bearing and sham-operated control animals were statistically significant (p<0.01).

There was no specific retention of the [^18^F]FEDL-derived radioactivity observed in other organs and tissues, except for kidneys, ureters and urinary bladder, which involved in normal physiologic clearance of this radiotracer. Clearance of [^18^F]FEDL-derived radioactivity from major organs and tissues followed the kinetics of blood clearance (**Fig. 2D,E**). Clearance of [^18^F]FEDL from the circulation exhibited a bi-exponential kinetics with half-lives of 1.65±0.50 min and 14.14±3.60 min, respectively (**Fig. 2D**). At 60 min post i.v. injection, the level of [^18^F]FEDL in blood was 0.51±0.24%ID/ml, determined from the maximum pixel activity within the ROI placed over the heart region. No accumulation of [^18^F]FEDL-derived radioactivity was detected in the skeletal structures up to 60 min post injection of [^18^F]FEDL. The biodistribution of [^18^F]FEDL-derived radioactivity in different organs and tissues at 60 min post i.v. injection is provided in **Table 1**.

**Table 1:** Radioactivity concentration (%ID/g) in different organs and tissues measured by PET/CT at 60 min post intravenous administration of [^18^F]FEDL.

Tissue	Average	St.Err.
Blood	0.51	0.11
Brain	0.18	0.05
Muscle	0.21	0.03
Kidney	5.14	2.47
Liver	0.66	0.23
Lung	0.77	0.59
Spleen	0.22	0.08
Stomach	0.20	0.12
Intestine	0.17	0.11
Pancreas	0.38	0.22
Peritumoral pancreas	4.04	2.06

### *In Vivo* [^18^F]FEDL Autoradiography and HIP/PAP Expression

**Figure 3:**
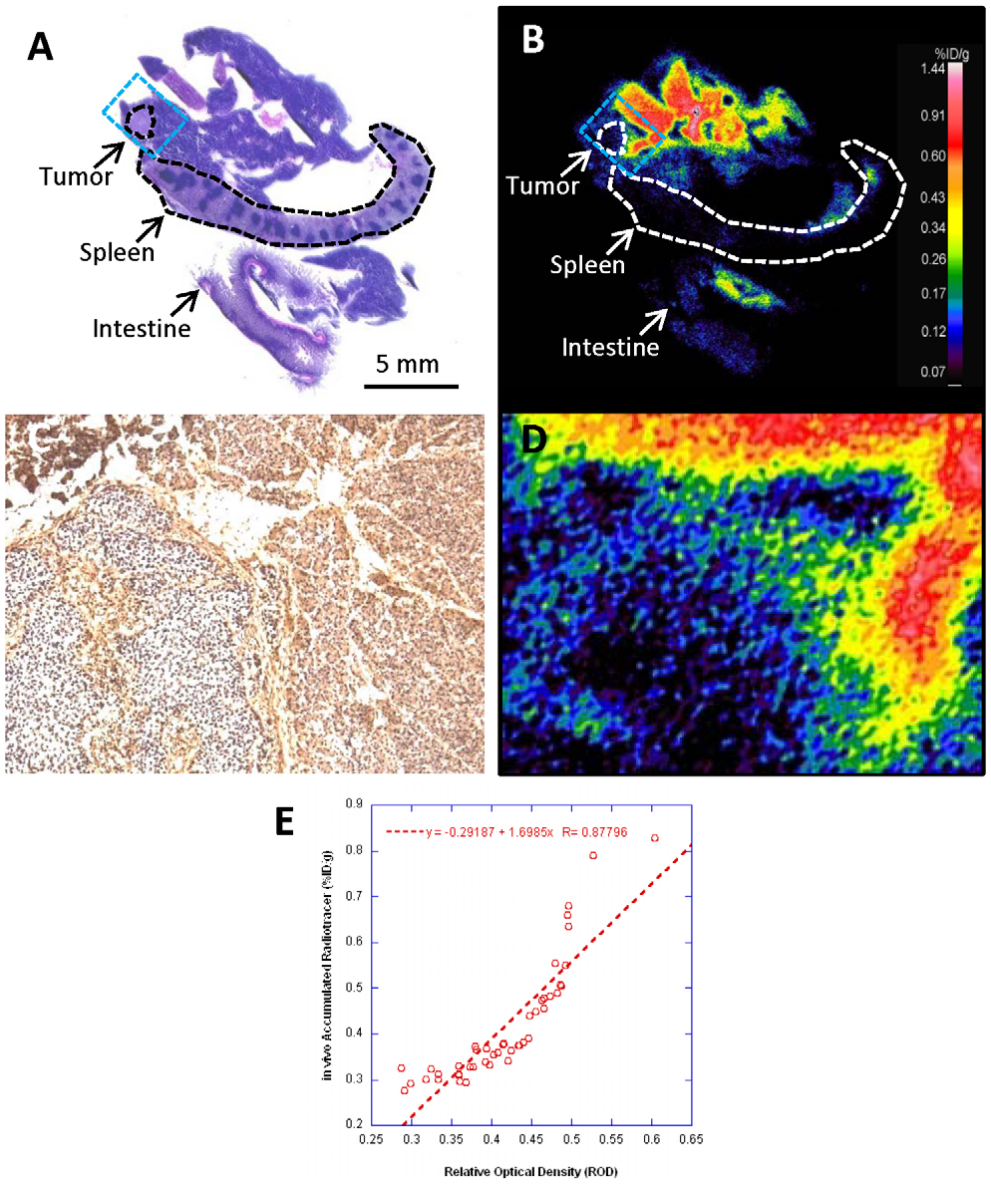
Autoradiography of [^18^F]FEDL distribution after intravenous administration and HIP/PAP expression. (**A**) H&E stained section and (**B**) color-coded autoradiographic image of [^18^F]FEDL-derived radioactivity distribution in an adjacent section obtained from the same tissue block including: pancreas, spleen and intestine and a small orthotopic pancreatic carcinoma lesion outlined by the dotted lines (bar: 5 mm) and indicated by the arrows; a blue dotted line outlines the area shown at ×15 higher magnification in panel (**C**) IHC of HIP/PAP and (**D**) autoradiography of [^18^F]FEDL-derived radioactivity distribution. High level of [^18^F]FEDL radioactivity is seen in the peritumoral pancreatic tissue, but not in the tumor lesion. (**E**) A scatter plot and linear regression analysis of relationship between the magnitude of HIP/PAP expression (IHC densitometry, ROD) and [^18^F]FEDL accumulation in peritumoral pancreatic tissue (autoradiography, PSL/mm^2^); an almost linear relationship is observed (r = 0.88).

*In situ* validation of *in vivo* [^18^F]FEDL PET/CT imaging was performed at the end of each dynamic imaging study using comparative analysis with autoradiography and immunohistochemistry of HIP/PAP expression in the pancreas. Distribution of [^18^F]FEDL-derived radioactivity in a block of tissues (**Fig. 3A**), including pancreas, spleen and a segment of intestine demonstrated high levels of [^18^F]FEDL binding accumulation in the peritumoral reactive pancreatic tissue (blue rectangle **Fig. 3B**, shown magnified in **3D**), which was consistent with the high level of HIP/PAP expression observed in peritumoral pancreatic tissue in the corresponding area (**Fig. 3C**). Comparative densitometric analysis of autoradiographic and IHC images demonstrated a good linear correlation (r = 0.88) between the magnitude of *in vivo* accumulation of [^18^F]FEDL and the level of HIP/PAP protein expression in different regions of peritumoral pancreas (**Fig. 3E**).

### *Ex Vivo* [^18^F]FEDL Autoradiography and HIP/PAP Expression

**Figure 4:**
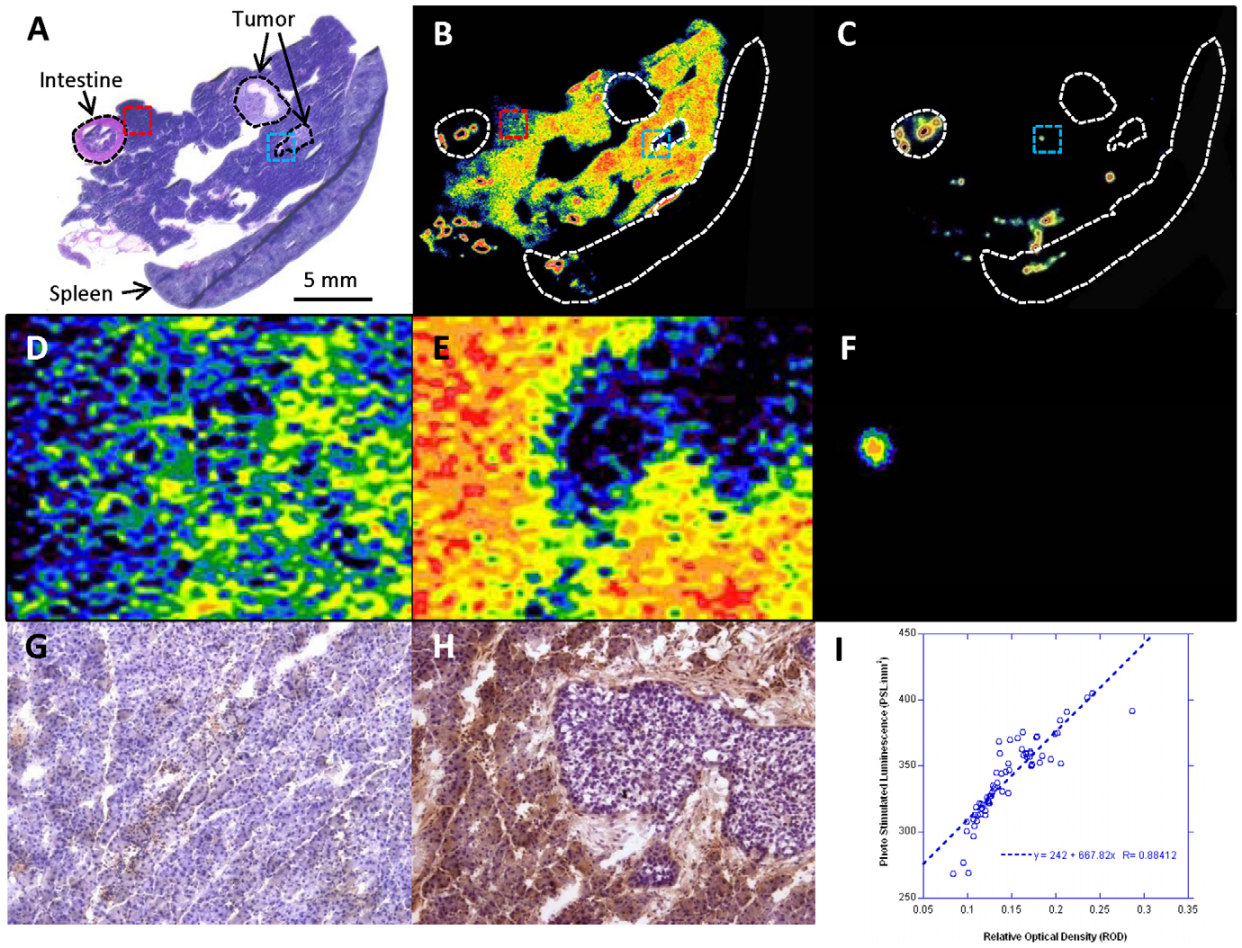
Comparison of *ex vivo* [^18^F]FEDL autoradiography and HIP/PAP expression. (**A**) H&E stained section and (**B**) color-coded autoradiographic image of [^18^F]FEDL binding in an adjacent section obtained from the same tissue block including: pancreas, spleen and intestine and two small orthotopic pancreatic carcinoma lesions outlined by the dotted lines (bar: 5 mm) and indicated by the arrows; a red dotted line outlines the area shown at ×15 higher magnification in panel **D** (autoradiography) and panel **G** (IHC) with low level of HIP/PAP expression; a blue dotted line outlines the area shown in panel **E** (autoradiography) and **H** (IHC) with high level of HIP/PAP expression. (**C**) autoradiographic image of an adjacent section blocked with 1mM lactose prior to exposure to [^18^F]FEDL. A non-specific binding is observed to the intestinal content and to the vascular- or ductal-appearing structures in the pancreras and spleen. (**F**) ×15 magnified autoradiographic image of the region outlined by the blue dotted rectangle in panel **C**, demonstrating some non-specific binding of [^18^F]FEDL. (**I**) A scatter plot and linear regression analysis of relationship between the magnitude of HIP/PAP expression (IHC densitometry, ROD) and [^18^F]FEDL binding (autoradiography, PSL/mm^2^); an almost linear relationship is observed (r = 0.88).

Additional evaluation of radioligand properties of [^18^F]FEDL was performed using comparative analysis of *ex vivo* autoradiography and HIP/PAP immunohistochemistry (IHC) (**Fig. 4**). This experiment was conducted using frozen pancreatic tissue sections obtained from mice bearing small orthotopic xenografts of L3.6pl/GL+ tumors (**Fig. 4A**). The autoradiographic images demonstrated a peritumoral pattern of [^18^F]FEDL binding to the reactive pancreatic tissue (blue rectangle **Fig. 4B**, shown magnified in **4E**), which was consistent with the high level of HIP/PAP expression observed in peritumoral pancreatic tissue in the corresponding area (**Fig. 4H**). In contrast, the magnitude of [^18^F]FEDL binding pancreatic tissue was significantly lower in the regions remote from the site of tumor growth (red rectangle **Fig. 4B**, shown magnified in **4D**), which was consistent with low (background levels of HIP/PAP expression in these remote regions of pancreas (**Fig. 4G**). Pre-treatment of an adjacent tissue section with 1 mM lactose resulted in a complete inhibition of specific binding of [^18^F]FEDL to pancreatic tissue (**Fig. 4C**). However, some non-specific binding was still observed, which had punctuate or small circular pattern the origins of which is, presumably, the intimal layer of larger vessels and intestinal content (blue rectangle **Fig. 4C**, magnified in **4F**). Comparative densitometric analysis of autoradiographic and IHC images demonstrated a good linear correlation (r = 0.88) between the magnitude of [^18^F]FEDL binding and the level of HIP/PAP protein expression in different regions (**Fig. 4I**).

## Discussion

In the current study, we have demonstrated that the detection of early stage pancreatic carcinomas is feasible using PET/CT with [^18^F]FEDL, a radioligand for HIP/PAP, which is selectively over-expressed in peritumoral pancreatic acinar cells. The extent of HIP/PAP over-expression in peritumoral pancreas resulted in a significant amplification of the apparent lesion size: at least a 2–4 fold amplification of the apparent tumor lesion size for sub-millimeter sized tumors to almost a half of the pancreas (10–12 mm) for tumors of 2–2.5 mm in diameter. The magnitude of peritumoral accumulation of [^18^F]FEDL was proportional to the level of HIP/PAP expression (r = 0.88), as demonstrated by a comparative densitometric analysis of QAR and IHC-stained tissues sections. It is noteworthy, that such relationship with HIP/PAP expression was observed using QAR after *in vivo* intravenous administration of [^18^F]FEDL (after PET/CT imaging) as well as using *ex vivo* QAR (after incubation of frozen tissue section in solution with [^18^F]FEDL). The specificity of [^18^F]FEDL binding was confirmed by pre-blocking of the tissue sections with excess of non-radiolabeled lactose. Together, these results further confirm the specificity of [^18^F]FEDL binding to the HIP/PAP protein-expressing peritumoral pancreatic tissue.

To quantify the magnitude of [^18^F]FEDL binding to peritumoral pancreatic tissue, we elected to utilize model-independent Logan graphical analysis, due to the limitations of repetitive blood sampling in mice, which hampers the radiolabeled metabolite analysis from small volumes of plasma. Instead of the arterial blood input function, the Logan graphical analysis utilizes time-activity data from a reference tissue [14]. Ideally, normal pancreatic tissue not expressing the HIP/PAP should be used as the reference tissue for peritumoral pancreatic tissue. However, the results of our studies using *in situ* QAR and IHC demonstrated in some cases a fairly extensive over-expression of HIP/PAP in peritumoral pancreas. Therefore, we concluded that the use of apparently normal pancreatic regions (not significantly accumulating [^18^F]FEDL) would be unreliable. In contrast, muscle tissue does not express HIP/PAP and was used as a reference tissue for the Logan graphical analysis. The calculated binding potential of [^18^F]FEDL in peritumoral pancreatic tissue using muscle as the reference tissue was 2.57±0.60, which was 20-fold higher than in the pancreas of sham-operated control animals.

No significant accumulation of [^18^F]FEDL-derived radioactivity was detected in the skeletal structures up to 60 min post injection of [^18^F]FEDL, which suggests that the radiotracer was not catabolized or de-fluorinated *in vivo*. The rapid whole body re-distribution and fast renal clearance of [^18^F]FEDL from circulation make this radiotracer especially suitable for imaging of the abdominal area, specifically of the pancreas and liver. A similar pattern of radioactivity biodistribution was previously reported for [^18^F]FDL in normal NMRI mice and in transgenic mice expressing β–galactosidase under the control of a ubiquitous ROSA-26 promoter [12]. The similarity in biodistribution of [^18^F]FEDL and [^18^F]FDL is not surprising, because the only difference between these radiotracers is that in the [^18^F]FDL, the 2′-position of glucopyranose moiety is directly labeled with ^18^F-fluoro group, while in the [^18^F]FEDL, the same 2′-position is derivatized with a ^18^F-fluoroethyl side chain. The low level of [^18^F]FEDL-derived radioactivity in the pancreas, spleen, liver and intestines at 60 min post i.v. injection may also enable the detection of hepatocellular carcinomas and cholangial carcinoma as well, because the HIP/HAP in known to be over-expressed by the tumor cells (but not the peritumoral liver) [15], [16].

Early detection of small pancreatic tumors will require screening of asymptomatic subjects for pancreatic cancer. Despite the demonstration of feasibility of [^18^F]FEDL PET imaging as a biomarker for detection of early pancreatic carcinomas in an experimental animal model, this diagnostic imaging approach alone can't be justified for pancreatic tumor screening. This diagnostic imaging approach, however, could be developed as a follow-up to other biomarker screening approaches that use readily available bodily fluids and tissues, such as blood, pancreatic juice obtained during endoscopic studies. Several genetic syndromes such as familial pancreatitis, Peutz-Jeghers syndrome, and familial atypical multiple mole melanoma are associated with an increased risk of pancreatic cancer, in addition to mutations in the tumor suppressor gene BRCA2 and several DNA mismatch repair genes [17]–[21]. Currently, genetic syndromes with a high incidence of pancreatic cancer are being targeted for screening [22], [23] using blood proteomic profiling [24], serum biomarkers, such as amyloid A [25], RCAS1 [26], CA 19-9, etc. Translational studies are underway to discover novel molecular biomarkers such as up-regulated genes and over-expressed proteins specific for pancreatic carcinomas that could be detected in serum and pancreatic juice. Among the DNA based biomarkers, K-ras mutations are present in 90% of pancreatic cancer. Therefore, other groups have been developing novel PET imaging agents targeting KRAS oncogene, such as the [^64^Cu]DO3A-PNA-peptide [27]. Both mRNA and protein levels of telomerase reverse transcriptase (hTERT) are significantly elevated in pancreatic juice of almost 90% of patients with pancreatic carcinomas [28], [29]. Several other proteins or protein fragments are up-regulated in pancreatic carcinoma cells and can be identified in blood (serum) using two dimensional gel electrophoresis or mass spectrometry or antibody-based methods. Recently, it was demonstrated that MUC1 is upregulated in blood of more than 90% of patients with pancreatic carcinoma [30]. The MUC-1 antigen is identified in pancreatic carcinomas and precursor lesions, but is not detected in normal pancreas. The overall sensitivity and specificity of ELISA for MUC-1 in serum were 82% and 85%, respectively, which is consistent with previous results. An exciting finding in their study is that 92% of stage I cancer cases were above cutoff value for positive response. A positive correlation was observed for mean concentration of MUC-1 in the serum with stage of disease. The same group has recently developed a bivalent ^111^In-DOTA-labeled antibody to MUC-1 and reported on successful gamma scintigraphy results in mice bearing s.c. xenografts of pancreatic carcinoma [31]. In the latter study, superior imaging results were obtained using the non-radiolabeled bivalent antibody for pretargeting of a radiolabeled TF-10 hapten peptide.

Serum levels of circulating HIP/PAP are also significantly elevated in pancreatic cancer the level of HIP/PAP correlates with tumor load, nodal involvement, distant metastasis and short survival [32], [33]. HIP/PAP protein was also elevated in pancreatic juice in patients with both pancreatic carcinoma and to the lesser magnitude in chronic pancreatitis and could be used to detect early pancreatic injury [34]. Therefore we suggest that ELISA measurements of serum HIP/HAP and MUC-1 levels could be added to a panel of tests for the routine screening of population to identify individuals at increased risk for the developing pancreatic carcinoma. Then, in HIP/HAP-positive individuals [^18^F]FEDL PET/CT imaging could be performed to identify the location of potential pancreatic carcinoma. Te [^18^F]FEDL PET/CT could then be followed by more invasive diagnostic imaging approaches, such as endoscopic sonography and endoscopic retrograde cholangio-pancreatography (ERCP), which have been successfully used to detect precancerous lesions in an autosomal dominant familial pancreatic cancer with high penetrance [22].

In summary, we have demonstrated that early stage pancreatic carcinomas could be detected with PET/CT with [^18^F]FEDL, a radioligand for HIP/PAP, which is selectively over-expressed in peritumoral pancreatic acinar cells. Molecular imaging of tumor-specific biomarkers, such as HIP/PAP, over-expressed in peritumoral reactive pancreatic tissue generates the “lesion size amplification” effect and improves the detectability of small pancreatic tumors that are otherwise poorly detectable by conventional non-invasive imaging modalities. When translated into the clinic, [^18^F]FEDL PET/CT imaging should enable earlier diagnosis of pancreatic carcinomas, guide additional diagnostic procedures, facilitate earlier surgeries and thus improve patients' survival.

## Materials and Methods

### Cell Culture and Gene Transduction

Human pancreatic cancer cells L3.6pl were kindly provided by Prof. Isaiah J. Fidler (UT-MD Anderson, Houston, TX). The L3.6pl cells were stably transduced with a retroviral vector bearing a dual reporter gene, termed GL, which is a tandem fusion of the green fluorescence protein and firefly luciferase genes. Transduced L3.6pl/GL+ cells were selected by FACS and characterized for fluorescence and bioluminescence properties as previously described [35]. The L3.6pl/GL+ cells were grown in flasks with DMEM/F12, supplemented with 10% fetal bovine serum, penicillin and streptomycin, at 37°C in a humidified atmosphere with 5% CO_2_. The cells were harvested by trypsinization; after centrifugation of cell suspension at 5000 rpm for 5 min the culture medium was aspirated, and the cell pellet was re-suspended in 10% heat inactivated serum obtained from nu/nu athymic nude mice (Charles River, Wilmington, MA) for ortothopic injection, as described below.

### Orthotopic Pancreatic Tumor Model

All studies in animals were performed according to protocol approved by the Institutional Animal Care and Use Committee of the UT MD Anderson Cancer Center. Four to six weeks old athymic nude mice (N = 8, nu/nu, Charles River, Wilmington, MA) were anesthetized by isoflurane inhalation (2–2.5% in oxygen). A small incision (2 cm) was made in the left abdominal wall. The spleen was then exteriorized along with the underlying pancreas and approximately 1×10^7^ of L3.6pl-GL+ cells suspended in mouse serum were slowly injected directly into the tail of the pancreas, as previously described [36]. Sham operated control group of animals (N = 8) received no injections into the pancreas to avoid non-specific inflammatory response, which could induce HIP/PAP expression in the pancreas around the site of injection. Then, the wound was closed with nylon sutures and treated with antibacterial, antimycotic cream. After the operation, the animals were warmed and monitored until conscious and then placed in HEPA-filtered cages with feed and water *ad libitum*.

### Bioluminescence Imaging

To monitor tumor development, the mice were given a single 150-mg/kg intraperitoneal dose of the D-luciferin (Xenogen,CA) on days 4, 7, and 10 after tumor implantation. Under the gas anesthesia (2% isoflurane in oxygen), the animals were placed into the light-tight chamber of the IVIS200 imaging system (Xenogen Corp., Almeda, CA). Bioluminescence images were acquired using the following parameters: image acquisition time 1–5 sec; binning 2; no filter; f/stop; field view, 10 cm. The signal intensity was quantified as sum of all detected photons within the region of interest per second per cm^2^ per steradian (ph/s/cm^2^/sr), using Living Image software (Xenogen Corp., Almeda, CA).

### Radiosynthesis of [^18^F]-FEDL

Detailed radiosynthesis procedure for [^18^F]-FEDL has been reported by us elsewhere [13]. Briefly, initial radiofluorination reactions were carried out using *β-O-D-galactopyranosyl-2,3,4,6-O-tetraacetyl-(1–4′)-[2′-trifluoromethanesufonyl-3′,6′,-O-dibenzoyl-1′-O-ethyl]-D-mannopyranose* precursor and Bu_4_N[^18^F]F in anhydrous acetonitrile at 80°C for 20 min to produce [^18^F]-FEDL. The crude product was passed through a silicagel SepPak cartridge and eluted with ethyl acetate. Then, the radiolabeled crude intermediate was purified using preparative HPLC. Then, the solvent was evaporated *in vacuo* and the radiolabeled intermediate was hydrolyzed with 0.5 M NaOMe in methanol at 80°C for 5 min. The final product was neutralized with 1N HCl. The purity of the [^18^F]-FEDL product was verified by TLC and HPLC.

### PET/CT Imaging

PET/CT imaging was performed using small animal PET/CT scanner INVEON (Siemens Preclinical Solutions, Knoxville, TN). Pancreatic tumor bearing mice (N = 8) were anesthetized with isoflurane (2% in oxygen) and their temperature kept at 38°C with a heat lamp. The CT imaging parameters were X-ray voltage of 80kVp, anode current of 500 µA, an exposure time of 300–350 milliseconds of each of the 360 rotational step. Dynamic PET imaging studies were acquired during 0–60 minutes after administration of [^18^F]-FEDL (3,7 MBq, in 100 µL of saline) intravenously as a slow bolus over1 min while the animals were positioned inside the gantry of the scanner. Images were reconstructed using two dimensional ordered subsets expectation maximization (OSEM) algorithm. PET and CT image fusion and image analysis were performed using software ASIPro 5.2.4.0 (Siemens Preclinical Solutions, Knoxville, TN).

### Image Analysis and Quantification of Receptor Binding Potential

Regional radioactivity concentrations (KBq/cm^3^ or µCi/cm^3^) were estimated from the maximum pixel intensities within regions of interest (ROIs) drawn around the tumor or organ on trans-axial slices of the reconstructed image sets. The radioactivity uptake in the peritumoral area of pancreas, in the heart and muscle were converted to percent of injected dose per gram of tissue (%ID/g). Logan graphical analysis [14] of the dynamic ROI data was used to assess the magnitude of [^18^F]-FEDL accumulation in the peritumoral pancreas. The ratio of integrated radioactivity concentration in peritumoral pancreas divided by the radioactivity concentration at given time point tumor uptake was set as the y-axis. The ratio of integrated reference tissue uptake divided tumor uptake was set s the x-axis of a Logan plot. The muscle as reference tissue because it has now HIP/PAP expression, while the possibility of HIP/PAP expression in remote regions of “normal” pancreas could not be excluded *a priori*. The slope of the linear portion of the Logan plot was distribution volume ratio (DVR). If metabolite corrected plasma curves are not available, the plasma curve can be replaced with reference region curve C_r_(t). Then the slope of the linear portion of the lot is DVR:
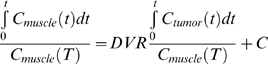
(1)


The binding potential (BP) can be calculated as:
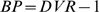
(2)


### Quantitative Autoradiography (QAR)

After PET imaging, the animals were sacrificed and pancreas with surrounding organs was rapidly extracted, frozen, and embedded in a mounting medium M1 (Shandon-Lipshaw, Pittsburg, PA). Serial 20 µm thick coronal sections of frozen tissues at were obtained at −13°C using a cryomicrotome (CM3050S, Leica, Germany). Tissue sections were thaw-mounted on poly-A lysine coated glass slides and heat-fixed for 5 min at 65°C on a slide warmer (Fischer Scientific, PA). For QAR, tissue sections were exposed to the phosphor plate (Fujifilm Life Science, Woodbridge, CT) along with set of 20 µm autoradiographic standards of known ^18^F radioactivity concentration, freshly prepared using calf liver homogenate. Knowing the radioactivity concentration in standards and the injected dose, the autoradiographic images were converted to color-coded parametric images of percent injected dose/g tissue (%ID/g) using MCID Elite image analysis software version 7.0 (Imaging Research Inc., St. Cathrines, Ontario, Canada).

For *ex vivo* QAR, 150 µl of saline containing [^18^F]-FEDL (240 µCi/15 ml) was gently applied directly onto glass slides containing frozen pancreatic tumor tissue sections with peritumoral pancreas, obtained from a separate group of tumor xenograft-bearing animals (not injected i.v. with the radiotracer). The tissue sections were placed in a humidified black box to reduce evaporation at room temperature for 60 min. The excess radioactivity was washed thrice by consecutive dipping in ice-cold PBS/Triton X-100 (0.01%) at pH 7.4. To assess the specificity of [^18^F]-FEDL binding, adjacent tissue sections were pre-blocked with cold lactose (1 mmol) for 30 min and then incubated with [^18^F]-FEDL, as described above. Both sets of slides were dried and exposed to phosphor plate (FLA5100, Fuji Photo Film Co., Tokyo Japan) for 6–8 hrs.

### Immunohistochemistry for HIP/PAP

The IHC for HIP/PAP was performed both in paraffin-fixed tissue sections and in frozen tissue sections, as required. Frozen sections (20 µm) or paraffin-embedded sections (5 µm thick) were incubated in 3% aqueous H_2_O_2_ to block the endogenous peroxidase activity, washed and then incubated in 2% normal horse blocking serum for 60 minutes at room temperature. Thereafter, the sections were incubated with monoclonal anti-mouse Reg II antibody (R&D System Inc., Minneapolis, MN) at 1∶50 dilution in Tris-NaCl overnight at 4°C. Then, the sections were incubated with the secondary biotinylated horse anti-mouse IgG, followed by avidin-peroxidase conjugation and 3′3-diaminobenzidine chromophore development steps using Vectastain ELITE kit (Vector Laboratories, Burlingame, CA) according the manufacturer's protocol. The tissue sections were either counterstained with hematoxylin or used without counterstaining for densitometric analysis of the intensity of HIP/PAP immunostaining.

### [^18^F]-FEDL Binding Relative to HIP/PAP Expression

Using MCID Elite software version 7.0 (Imaging Research Inc., St. Cathrines, Ontario, Canada), the IHC-stained tissue sections were digitized and co-registered with the corresponding adjacent QAR images. The radioactivity concentration (%ID/g) in QAR images was quantified in ROIs as described above. For ex vivo autoradiography, the phosphor plate image intensity was quantified as units of photo-stimulated luminescence per square mm (PSL/mm^2^). The intensity of immunostaining for HIP/PAP in corresponding ROIs in adjacent tissue sections was quantified by densitometry and expressed as relative optical density (ROD). The data was plotted against each other and linear regression analysis performed to assess the relationship.
